# The impact of thyroid disorder on cardiovascular disease: Unraveling the connection and implications for patient care

**DOI:** 10.1016/j.ijcha.2024.101536

**Published:** 2024-10-23

**Authors:** Nanny Natalia Mulyani Soetedjo, Dessy Agustini, Hikmat Permana

**Affiliations:** a Division of Endocrinology, Metabolism, and Diabetes, Department of Internal Medicine, Faculty of Medicine, Padjadjaran University, Bandung, West Java 45363, Indonesia; b Faculty of Medicine, Universitas Sriwijaya, Palembang, South Sumatra 30114, Indonesia

**Keywords:** Thyroid hormone, Cardiovascular disease, Hyperthyroidism, Hypothyroidism

## Abstract

The thyroid gland is responsible for metabolism, as well as cardiac function and the peripheral vascular system. Thyroid dysfunctions are associated with an increase in the risk of cardiovascular diseases, including heart failure and coronary heart disease atrial fibrillation, by impairing heart contractility, stroke volume, heart rate, peripheral vascular resistance, and electrical activity. Thyroid dysfunctions also alter several cardiovascular risk factors, such as atherosclerosis, hypertension, and dyslipidemia, as well as causing stroke, which is associated with atrial fibrillation. An antiarrhythmic drug, amiodarone, may also induce both thyrotoxicosis and hypothyroidism, so its use requires serial thyroid function testing. Every CVD patient is recommended to be screened and treated for any possible thyroid dysfunction to reduce the patient’s mortality and morbidity.

The thyroid gland is a vital endocrine organ responsible for producing two primary hormones: triiodothyronine (T3) and tetraiodothyronine (T4; thyroxine) [1]. They are not only necessary for sustaining metabolism, but also controlling cardiac function and the peripheral vascular system [2]. Thyroid hormone receptors (THRs) are present in blood vessels and heart tissues, which means that changes in the levels of thyroid hormone in the bloodstream can affect the functioning of these organs [3]. It is generally documented that they increase cardiac contractility and heart rate, improve systolic and diastolic function, and decrease systemic vascular resistance (SVR) under resting conditions. As a result, thyroid alterations are related to an increase in the risk of developing cardiovascular disease (CVD) [4].

Patients who exhibit prominent indications of hypo- or hyperthyroidism have well-established symptoms related to the cardiovascular (CV) system and blood. Neglecting these symptoms can accelerate the development of symptomatic CVD [3]. It can potentially induce or worsen CV issues such as irregular heart rhythms in the atria and ventricles, blockage of arteries due to plaque buildup, abnormal serum lipid levels, and heart failure (HF). These alterations increase the likelihood of experiencing health problems and premature mortality. Moreover, an increasing amount of observational evidence indicates that some patient subgroups with subclinical thyrotoxicosis or hypothyroidism may have an elevated risk of CV problems. Specifically, subclinical thyroid dysfunction has been linked to a 20 % to 80 % higher incidence of vascular morbidity and mortality [3], [5]. Therefore, this review seeks to investigate the existing understanding of the influence of thyroid disorders on CV disease, as well as the implications to patients’ care.

## 1 A glimpse of thyroid hormone and its effects on cardiovascular system

**Fig. 1:**
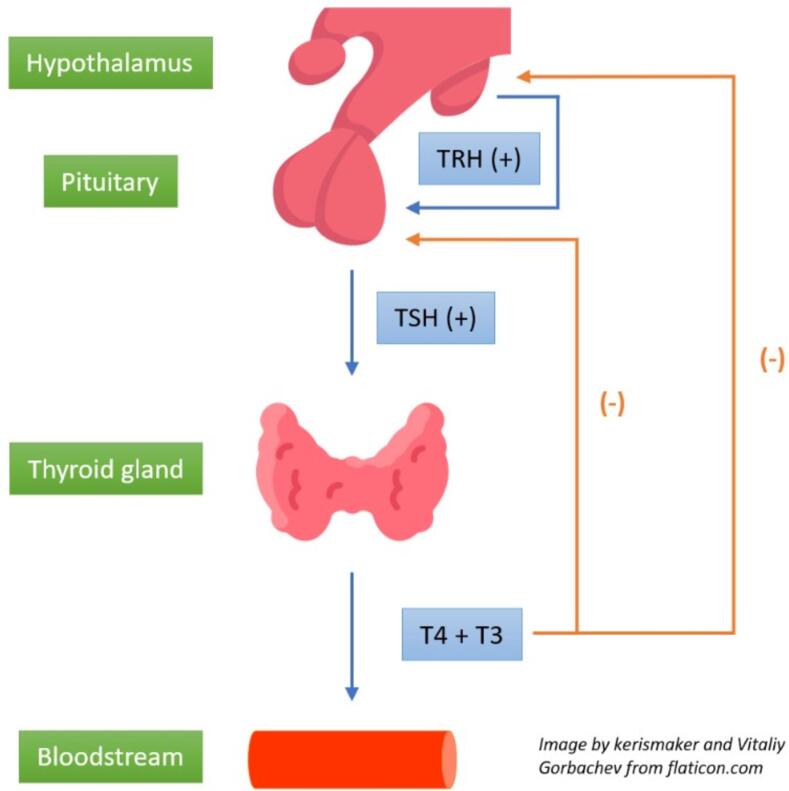
Feedback mechanism in hypothalamic-pituitary-thyroid axis, adapted from Fig. 1 of reference number [7].

Thyroid hormones (THs) are vital for the optimal development and functioning of various human tissues. Additionally, they are essential for the metabolic regulation of all cells and organs in the human body throughout one’s lifespan [6]. The hypothalamic-pituitary-thyroid axis is a feedback loop which is responsible for regulating thyroid function (Fig. 1) [7]. The hypothalamus releases thyrotropin-releasing hormone (TRH), which triggers the anterior pituitary gland to produce thyroid-stimulating hormone (TSH). TSH stimulates the thyroid gland, prompting it to release TH. TH levels control the release of TRH and TSH. Even slight alterations in thyroid hormone concentrations result in significant fluctuations in TSH levels. Thus, serum TSH serves as a dependable marker for assessing the overall thyroid hormone status in the body. Thyroxine (T4) and triiodothyronine (T3) are the main iodinated thyroid hormones. Both T3 and T4 possess biological effects. However, T3 is regarded as an active and more potent hormone [3].

**Fig. 2:**
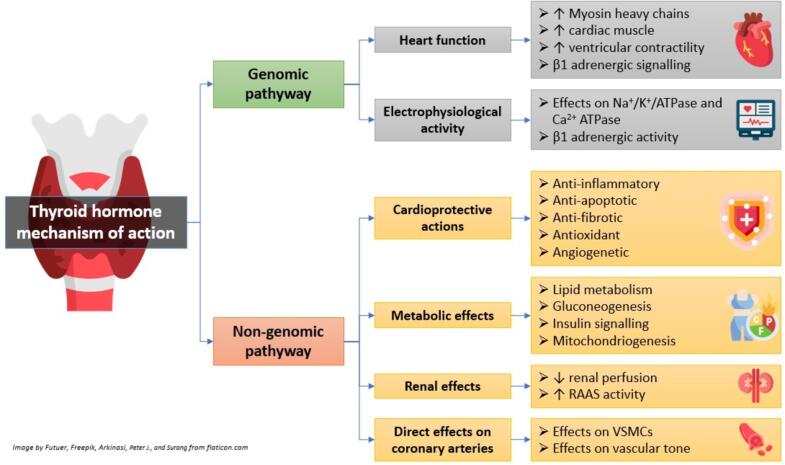
Effects of thyroid hormones on cardiovascular system, adapted from Fig. 1. of reference number [8].

Thyroid hormones also influence the autonomous nervous system and the renin-angiotensinogen-aldosterone system through genomic and non-genomic mechanisms which affect the circulatory system, as shown in Fig. 2 [8]. The most recognized modes of action of THs depend on direct effects on transcription control, facilitated by nuclear receptors (genomic mechanism) [9]. The genomic effects of TH play a recognized role in the development, differentiation, and homeostatic regulation of target tissues [10]. However, the non-genomic effects of TH manifest swiftly and are uninfluenced by transcription inhibitors and protein synthesis [9].

There are two ways in which thyroid hormones work: first, in the nucleus, where they bind to specific receptors that bind to specific elements in the promoters of genes that the hormones are targeting (genomic actions); second, rapidly outside of the nucleus, in the blood vessels and cardiac myocytes, where they have an effect that does not involve transcription regulated by transcription factors (non-genomic actions). The cytoplasmic organelles and plasma membrane are quickly impacted by these actions. Transcription and translation inhibitors have no effect on the many fast effects facilitated by these hormones. On the other hand, T3-mediated effects include alterations in intracellular signaling pathways in cardiac and vascular smooth muscle cells, changes in actin polymerization, and alterations in various membrane sodium, potassium, and calcium ion channels [10]. Consequently, they possess both electrophysiological (chronotropic) and contractile (dromotropic) properties, which impact the contractility, structure, and electrophysiological activity of the myocardium [8].

Thyroid hormones affect cardiac function by acting on genomic pathways within cardiomyocytes via binding to nuclear receptors, which regulate target genes’ expression [11]. The binding of T3 to TH receptor (THR) increases several cardiac genes expression that are contributed in the heart’s ability to contract, including sarco/endoplasmic reticulum free calcium ATPase 2 (SERCA2a), voltage-gated potassium channels, Na^+^/K^+^ ATPase, α-myosin heavy chains, and β1-adrenergic receptor adenine nucleotide translocase [12]. Extranuclear/non-genomic pathways have various impacts on the cardiomyocyte’s ion channels and peripheral circulation, which regulate hemodynamics, myocardial contractility, and heart-filling [11]. Vascular smooth muscle cells (VSMCs) are directly impacted by T3, inducing relaxation. T3 furthermore stimulates the phosphoinositol 3-kinase (PI3-K)/Ak-mediated signaling pathway of endothelial nitric oxide synthase, enhancing the nitric oxide (NO) production. NO exerts a vital role in regulating the equilibrium of blood vessels, reducing the constriction of blood vessels [13].

An overabundance of thyroid hormones is the hallmark of hyperthyroidism. Overt hyperthyroidism is specified by decreased serum TSH and increased free T4 (FT4) in serum, whereas subclinical hyperthyroidism (SCHyper) is distinguished by lower serum TSH levels and normal TH levels [8], [11]. The predominant forms of hyperthyroidism in iodine-sufficient and iodine-deficient regions are Graves’ disease and toxic nodular goiter, respectively [14]. Type 1 refers to TSH levels between 0.1 and 0.4 mIU/L, while type 2 denotes TSH levels less than 0.1 mIU/L. Given the different ways in which these two groups might progress to overt hyperthyroidism and cause harm to the body, it’s crucial to distinguish between them. Approximately 7 % of people develop overt hyperthyroidism each year, whereas 12 % have their levels return to normal. A reduced TSH level enhances the likelihood of advancement and diminishes the probability of spontaneous normalization [15].

Conversely, hypothyroidism is a medical condition characterized by inadequate synthesis of thyroid hormonesWhile elevated TSH and decreased T4 indicate overt hypothyroidism, elevated TSH and normal T4 and T3 levels indicate subclinical hypothyroidism (SCHypo). Grade 1 SCHypo is defined as TSH values between 9.9 mIU/L and the upper normal limit, and grade 2 is defined as TSH levels of 10 mIU/L or higher. Grade 1 may be further categorized into patients with TSH levels below 7 and those between 7.0 and 9.9 mIU/L in younger individuals (<65 years) to assess the efficacy of levothyroxine medication [16]. Low T3 syndrome (LT3S), commonly called nonthyroidal sickness syndrome or euthyroid sick syndrome, is marked by a decline in both total and free T3 levels. In contrast, levels of T4 and TSH in the blood remain within the normal range, and rT3 levels increase [11].

## 2 Causes of thyroid disorders

### 2.1 Hyperthyroidism

Graves’ disease is the predominant etiology of hyperthyroidism, an autoimmune disorder characterized by autoantibodies targeting the thyroidal TSH receptor, resulting in augmented production and release of thyroid hormones [17]. The second most common cause is toxic nodular disease, which affects 1.5–18 cases per 100,000 person-years worldwide. It is characterized by thyroid gland nodules releasing excess thyroid hormone and is more common in iodine-deficient locations [17], [18]. Certain medications, including amiodarone and immune checkpoint inhibitors for certain cancers, together with excessive thyroid hormone prescriptions, might induce hyperthyroidism [18]. Uncommon causes of thyrotoxicosis include painless and subacute thyroiditis, resulting from inflammation of thyroid tissue that leads to the release of preformed hormones into the bloodstream [19].

In order to develop a suitable treatment strategy, it is crucial to evaluate thyrotoxic symptoms, paying particular attention to possible cardiovascular and neuromuscular consequences [19]. TSH, FT4, FT3, and thyroid antibodies, particularly thyroid receptor antibodies (TRAb), should all be tested routinely to discover the cause of thyrotoxicosis. Thyroid uptake scans are beneficial when the clinical characteristics and blood tests do not provide a firm diagnosis. Thyroid sonography has a limited role in the evaluation of patients with thyrotoxicosis and is not required as part of routine testing [20].

### 2.2 Hypothyroidism

Hypothyroidism may result from inadequate synthesis and secretion of thyroid hormones or from impaired TRH or TSH signaling to the thyroid [21]. Central hypothyroidism is often associated with hypothalamo-pituitary diseases and other pituitary hormone dysfunctions. The primary causes of hypothyroidism are significantly affected by environmental conditions, resulting in considerable variability in its worldwide distribution. In regions lacking iodine, this is the principal cause of hypothyroidism, whereas autoimmunity is the predominant factor in iodine-replete areas [22]. In the United States and other iodine-abundant regions, chronic thyroiditis, referred to as Hashimoto thyroiditis, is the predominant cause. Central hypothyroidism has a far lower incidence, estimated at 1 in every 1000 cases of hypothyroidism, and is mostly attributed to a pituitary adenoma. This is a common side effect of therapy for many disorders, affecting 20 % to 50 % of individuals undergoing radiation for nasopharyngeal and paranasal sinus cancers [23].

Iodine, a naturally occurring trace element, is absorbed via the small intestine and is present in fish, seaweed, vegetables, and is supplemented in table salt [24]. Iodine shortage leads to thyroid insufficiency, since the thyroid requires iodine to synthesize T4 and T3. Transient hypothyroidism may occur due to excess iodine, referred to as the Wolff-Chaikoff effect [23]. Excessive iodine consumption might result in the synthesis of iodopeptides that obstruct thyroid peroxidase (TPO) mRNA, causing a reduction in the Na^+^/I^-^ symporter over many weeks until baseline intrathyroidal iodine levels are restored [24].

When the immune system incorrectly targets healthy thyroid proteins including thyroglobulin (Tg) and TPO, a condition known as Hashimoto thyroiditis develops. This is the most prevalent type of autoimmune thyroid disease (AITD). The presence of anti-TPO antibodies in the bloodstream can be used to diagnose autoimmune thyroid disease (AITD) with a sensitivity of 90 %. This condition affects 80 to 90 % of affected persons. Consequently, these antibodies cause thyrocyte fibrosis and lymphocytic infiltration of the thyroid [24]. The diagnosis of autoimmune thyroid disease (AITD) is contingent upon various factors: the presence of circulating antibodies targeting the thyroid; hypoechogenic and heterogeneous gland parenchyma observed via ultrasonography; and elevated thyroid stimulating hormone (TSH) levels, accompanied by normal or low serum thyroid hormones in a subset of patients [25].

Hypothyroidism has been associated with a number of medications, including thionamides, amiodarone, lithium, tetracyclines, and even several antineoplastic medications. Thionamides, suppress TPO activity, resulting in a therapeutically beneficial impact in the management of hyperthyroidism [24]. Amiodarone has a significant iodine content, which may induce hyperthyroidism or suppress thyroid function, thereby leading to hypothyroidism [21]. Lithium has a concentration in the thyroid that is 3 to 4 times larger than that in plasma, where it might impede iodotyrosine coupling and obstruct the release of thyroid hormones. Finally, patients commencing beta blocker medication must be examined for indications of hypothyroidism, since beta blockers may impede the deiodination of T4 to T3 and enhance the conversion to inactive rT3 [24].

## 3 Thyroid alterations in patients with heart failure, atrial fibrillation, and coronary heart disease

Individuals with even slightly modified thyroid function experience a more unfavorable outlook in heart disease, including HF [26]. Hypothyroidism can impair myocardial contractility, particularly during the relaxation phase, as well as inhibit cardiac muscle relaxation. Myocardial diastolic function is influenced by both related diastolic hypertension and, in some situations, coexisting coronary heart disease (CHD). Considering that T3 plays a crucial role in controlling gene expression in the heart muscle, this decrease is anticipated to impact the contraction and remodeling of the myocardium. Reduced levels of free T3 have also been linked to higher mortality rates in individuals with heart disease [27]. According to a *meta*-analysis conducted by Wang (2017), the combined LT3S prevalence in CVD patients reached 17.6 % (95 % CI = 14.5 %–21.2 %). The prevalence is higher in heart failure (17.9 %; 95 % CI = 11.8 %–26.1 %), acute myocardial infarction (17.5 %; 95 % CI = 5.9 %–41.9 %), and acute coronary syndrome (ACS) patients (12.1 %; 95 % CI = 9.0 %–16.2 %) [28]. A recently published *meta*-analysis of 19,310 patients with CHD revealed that people with LT3S and hypothyroidism had a higher chance of dying from any cause and having severe adverse cardiovascular events compared to those without these conditions. Similarly, ACS patients were at a higher risk of cardiovascular events when suffering LT3S or hypothyroidism [29].

In addition, short-term hyperthyroidism is marked by increased cardiac output, resulting in considerably raised heart rate, contractility, and cardiac preload but reduced peripheral vascular resistance, causing a hyperdynamic circulation [11], which in turn leads to a significant increase in cardiac output [26]. It may also alter venous return and vascular resistance via the relaxation of VSMCs, resulting in circulatory congestion. The renin-angiotensinogen-aldosterone system is stimulated, which causes fluid and sodium retention [8]. Persistent hyperthyroidism can have a negative impact on heart’s structure and function because it has the potential to increase the size of the left ventricle, stiffen the arteries, enlarge the left atrium, and impair the left ventricle’s ability to relax and fill during the diastolic phase, resulting in a decline in left ventricle performance, which leads to HF [11]. A *meta*-analysis of 7 trials comprising 31,138 patients found that overt hyperthyroidism is associated with a 13 % increase in overall mortality and a 21 % increase in death from CVD [30]. A recently published *meta*-analysis, comprising 37 studies and involving 113,393 individuals with hyperthyroidism, has demonstrated that overt hyperthyroidism significantly raises the likelihood of developing CHD, stroke, and cardiovascular mortality [31].

Hyperthyroidism is linked to increased supraventricular ectopic activity, which can lead to heart arrhythmias, such as atrial fibrillation [8]. Individuals aged 60 or older who took part in the Framingham Heart Study and had a TSH level of 0.1 mIU/L or lower had a 3.3-fold higher chance of developing atrial fibrillation. Additional analysis of the Cardiovascular Health Study indicated that patients aged ≥ 65 with low TSH (<0.45 mIU/L) had twice the risk of developing atrial fibrillation, even if their free T4 levels fell within the normal range. Even people with TSH levels ranging from 0.1 to 0.44 mIU/L had a 1.85-fold increased risk [5].

## 4 Thyroid alterations in patients with atherosclerosis, hypertension, and dyslipidemia

Thyroid dysfunction can modify various cardiovascular risk factors, including atherosclerosis, hypertension, and dyslipidemia. Carotid intima medium thickness (CIMT) can be a dependable and noninvasive indicator of atherosclerosis in individuals with SCHypo [32]. A recent *meta*-analysis of 12 clinical studies found that administering thyroid medication to people with SCHypo significantly reduced CIMT levels, improved lipid profiles, and an inhibited atherosclerosis progression [33]. Results from CMIT and SCHyper research are inconsistent. Some studies have found no correlation; however, others have discovered that SCHyper has thicker CIMT. Low serum TSH and high plasma fibrinogen may enhance CVD risk. SCHyper is linked to CVD (CHD, HF, AF) and CV mortality [26].

Both hyper and hypothyroidism raise blood pressure. Hyperthyroidism raises systolic arterial pressure and frequently results in pulmonary artery hypertension (PAH), causing right ventricular dysfunction [26], [34]. Hypothyroidism is characterized by increased stiffness of the arteries and reduced activity of renin, which contribute to vascular dysregulation and elevated blood pressure. The absence of the typical vasodilator effects of T3 is the cause of this alteration [11]. A recent *meta*-analysis examined how levothyroxine replacement medication affects blood pressure in SCHypo. Systolic blood pressure dropped considerably after levothyroxine medication in 10 randomized clinical trials. Systolic and diastolic blood pressure dropped substantially following levothyroxine beginning in 19 prospective follow-up studies [35].

Thyroid hormone modifies the lipid profile, particularly by reducing cholesterol levels in the bloodstream. Thus, people suffering from hypothyroidism exhibit heightened levels of total and low-density lipoprotein (LDL), along with raised apolipoprotein B levels [36]. Overt hypothyroidism can lead to lipid profile changes, causing altered LDL, B apolipoprotein, C-reactive protein (CRP), and homocysteine. It can also cause dysfunction in endothelial function and coagulation activity [11]. Currently, there is a link between Grade 2 (severe) SCHypo (TSH levels > 10 mIU/L) and hyperlipidemia [37]. A *meta*-analysis of 35 case-control and cohort studies revealed that patients with Grade 1 (mild) SCHypo (serum TSH levels <10 mIU/L) exhibited notably elevated levels of total cholesterol, LDL, and triglycerides. Additionally, they displayed markedly reduced amounts of high-density lipoprotein (HDL) in comparison to people with normal thyroid function [38].

## 5 Thyroid alterations in patients with stroke

Hyperthyroidism is highly related to AF, which increases the risk of mortality and stroke [39]. Jiang (2017) conducted a *meta*-analysis of 11 trials with 3,936 patients. Lower T3 levels were linked to a poor prognosis in acute ischemic stroke [OR = 0.27, 95 % CI = 0.09–0.85, P = 0.02]. Patients with a poor prognosis exhibited considerably higher levels of FT4, while free T3 (FT3), total T3, and FT3/FT4 ratio were low. In a therapeutic stress situation, T4 to T3 conversion may be slowed. Consequently, the T3 level is expected to decrease while the T4 level increases. In contrast, population-based research has indicated that hyperthyroidism increases the chance of having an ischemic stroke, having a worse outcome after a stroke, and higher mortality rate. The researchers also discovered that pre-existing hyperthyroidism has thyrotoxic effects on ischemic brain tissue [40].

The summarized effects of thyroid dysfunctions on cardiovascular disease can be seen on Table 1.

**Table 1:** Current evidences of thyroid dysfunctions on cardiovascular diseases.

**Author**	**Main findings**
**Hyperthyroidism**	
Brandt (2011) [30]	Overt hyperthyroidism is associated with a 13% increase in overall mortality and a 21 % increase in death from CVD (RR = 1.21, 95 % CI 1.05–1.38).
Sohn (2020) [31]	Overt hyperthyroidism significantly raises the likelihood of developing CHD (HR = 1.47, 95 % CI 1.03–1.19), stroke (HR = 1.35, 95 % CI 1.03–1.75), and cardiovascular mortality (HR = 1.24, 95 % CI 1.07–1.45).
Jiang (2017) [40]	Acute ischemic stroke patients with higher levels of FT4 were at risk of poor prognosis (OR = 1.06, 95 % CI 1.01–1.10, P = 0.01).

**Hypothyroidism**	
Wang (2017) [28]	• Combined LT3S prevalence in CVD patients reached 17.6 % (95 % CI = 14.5 %–21.2 %). Higher prevalence in heart failure (17.9 %; 95 % CI = 11.8 %– 26.1 %), acute myocardial infarction (17.5 %; 95 % CI = 5.9 %–41.9 %), and acute coronary syndrome (ACS) patients (12.1 %; 95 % CI = 9.0 %–16.2 %). LT3S was associated with increased risk of all-cause mortality in cardiovascular patients (HR = 2.49, 95 % CI 2.04–3.03, P < 0.001; I^2^ = 52.6 %).
Chang (2020) [29]	• Ischemic heart disease population with hypothyroidism was associated with higher risk of all-cause mortality (HR = 1.47, 95 % CI 1.10–1.97). and MACE (HR = 1.53, 95 % CI 1.19–1.97). Ischemic heart disease population with LT3S was associated with higher risk of all-cause mortality (HR = 2.61, 95 % CI 1.89–3.59). and MACE (HR = 2.22, 95 % CI 1.71–2.89). In subgroup analysis, ACS patients with LT3S were associated with higher risk of all-cause mortality (HR = 3.30; 95 % CI = 2.43–4.48) and MACE (HR = 2.19; 95 % CI = 1.45–3.30).
Aziz (2017) [33]	• Treatment with thyroxin in subjects with SCHypo significantly decreased carotid intima medium thickness [WMD −0.32; 95 % CI (−0.47 to −0.16), P = <0.0001; I^2^ = 2 %]. Treatment with thyroxin in subjects with SCHypo significantly decreased total cholesterol, triglyceride, low-density lipoprotein, sistolic blood pressure, and diastolic blood pressure.
Jiang (2017) [40]	Lower total T3 levels were negatively associated with a poor prognosis in acute ischemic stroke [OR = 0.27, 95 % CI = 0.09–0.85, P = 0.02].

## 6 Thyroid alterations in patients treated with amiodarone

Clinicians should pay attention to the use of amiodarone, a potent antiarrhythmic drug, since it may cause amiodarone-induced thyrotoxicosis (AIT) and amiodarone-induced hypothyroidism (AIH) [34]. Amiodarone has dual effects on the myocardium as it acts as an ion channel inhibitor and β-blockers. Additionally, it shares a similar structure with T3 and may act as an antagonist at THRs. Amiodarone is a selective inhibitor of deiodinases, which prevents the conversion of T4 to T3, causing functional hypothyroidism. Amiodarone inhibits TH activity and blocks peripheral functions, enhanced by the suppression of organification in the thyroid gland due to a high intake of iodine, known as the Wolff-Chaikoff effect [41]. AIH manifests as primary hypothyroidism. Therefore, the diagnosis and treatment are similar, including levothyroxine medication [27].

**Fig. 3:**
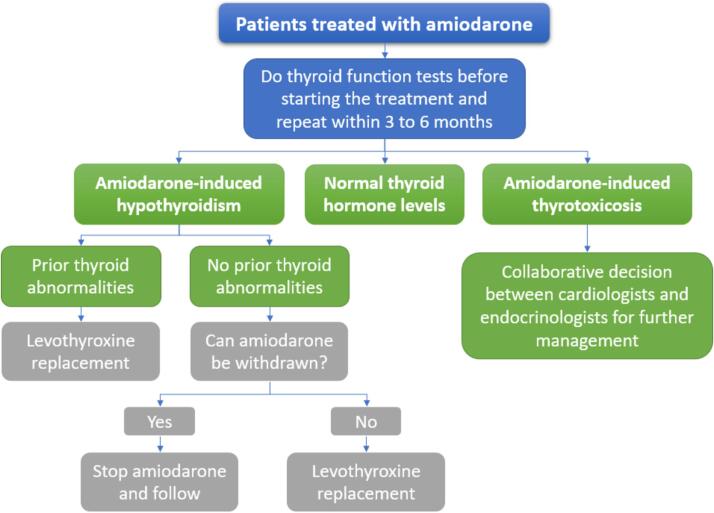
Management of amiodarone-induced thyroid alterations [27], [34], [41].

In other ways, AIT can occur either due to an excessive amount of iodine, leading to a temporary increase in TH production (type I), or due to iodine-induced toxicity, which leads to thyroiditis and elevated release of TH (type II) [41]. However, existing methods to differentiate between these types could be better, as some patients show similarities between the two types. There is a need for new markers that can circulate in the body and imaging modalities for the thyroid that can provide more accurate and timely differential diagnosis [5]. The working group suggests conducting thyroid function tests before starting amiodarone and repeating them within 3 to 6 months after starting the medicine, as long as there are no indications of thyroid problems [41]. The treatment summary for amiodarone-induced thyroid alterations can be seen in Fig. 3.

## 7 Diagnosing and managing thyroid dysfunction related to cardiovascular disease

**Fig. 4:**
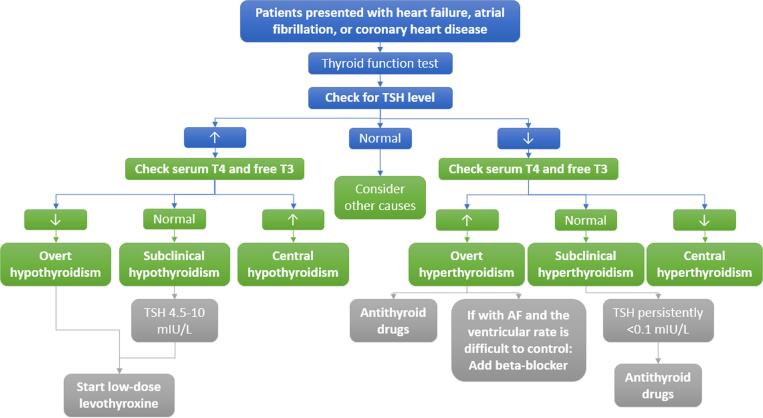
Summary of thyroid function screening in patients with cardiovascular disease.

Most worldwide recommendations recommend the thyroid function test as a class I indication for all patients with HF and CHD [34]. Thyroid function tests are used to evaluate the thyroid status by measuring them in peripheral blood. Existing assays facilitate the measurement of the quantities of TSH, as well as the T4 and T3 [5]. Table 2 displays the strategies for managing thyroid dysfunction with CVD [34], while the summary of the thyroid screening algorithm in patients with CVD can be seen in Fig. 4.

**Table 2:** Managing the comorbidity of thyroid dysfunction with CVD [34].

**Conditions**	**Managing thyroid dysfunction**	**Managing cardiovascular symptoms**
Heart failure, dilated cardiomyopathy	• At initial presentation, do thyroid testing using the established guidelines. Elderly individuals with cardiac problems or severe hypothyroidism should start with a low dose of levothyroxine (25–50 μg/day) and adjust every 10–12 weeks to maintain normal TSH levels.	• Volume control: diuretics Hyperadrenergic state control: β-blockers
Atrial fibrillation, supraventricular tachycardia	• At initial presentation, do thyroid testing Antithyroid drugs, as per the guidelines, are used to manage hyperthyroidism	β-blockers are the preferred treatment for controlling heart rate. If they cannot be used due to contraindications, calcium channel antagonists such as diltiazem or verapamil are used instead.
Amiodarone	• Thyroid testing should be done before starting the medication, within 3 months of starting, and every 3–6 months thereafter for patients with AIH. In cases of obvious AIH, treatment with levothyroxine is recommended. However, in some mild cases, especially in older individuals, levothyroxine treatment may be avoided. In such cases, regular thyroid function monitoring is necessary to detect any potential development of overt hypothyroidism. Patients with AIT should be recognized as being at high risk for emergency medical intervention, especially in older individuals and those with impaired left ventricular function. These patients should be treated according to established treatment protocols.	• If amiodarone considered essential for the underlying heart condition, AIH does not require its withdrawal. The choice to either continue or discontinue amiodarone in AIT should be personalized based on cardiovascular risk assessment and made collaboratively by cardiologists and endocrinologists.

Acronym: TSH – thyroid-stimulating hormone; CVD – cardiovascular disease; AIH–amiodarone-induced hyperthyroidism; AIT – amiodarone-induced thyrotoxicosis.

Antithyroid medicines are the recommended treatment for hyperthyroidism. It is essential to regularly monitor the thyroid profile every 4–8 weeks after starting therapy and make necessary adjustments to the dosage. Administration of potassium iodide orally or sodium iodide intravenously, in conjunction with antithyroid medications, is recommended to decrease the secretion of preexisting thyroid hormone reserves within the gland. Thyroidectomy is the ideal treatment for individuals who cannot tolerate antithyroid medications or ^131^I ablation and for those who experience compressive symptoms due to a nodule or goiter [34]. Based on the 2016 American Thyroid Association guideline, When the TSH level remains consistently below the normal range but is greater than or equal to 0.1 mIU/L, patients aged 65 and older, as well as those with heart conditions, osteoporosis, or hyperthyroidism symptoms, may benefit from considering SCHyper medication. To rule out temporary thyroiditis, a TSH level must remain between 0.1 and 0.4 mIU/L on multiple measurements taken 3 to 6 months apart. In order to treat SCHyper, one must first determine the cause of the thyroid dysfunction. Then, one must adhere to the guidelines as when treating overt hyperthyroidism [19]. When the TSH level is <0.1 mIU/L, it is recommended to repeat the testing of TH levels and antithyroid antibodies in the span of two weeks. If the TSH level is between 0.1–0.45 mIU/L, the retesting should be done within 1–3 months [34].

β-blockers and diuretics are taken to regulate heart rate, alleviate adrenergic symptoms in patients with hyperthyroidism, as well as enhance the management of persistent tachycardia and heart failure (HF). It is recommended to manage adrenergic symptoms with propranolol, 20–40 mg every 6 hours, or with longer-acting β-blockers like bisoprolol and atenolol. Propranolol in high doses (40 mg every 6 hours) hinders the peripheral conversion of T4 to T3. The preferred initial treatment for tachycardia in thyroid storm should be β1-selective adrenergic receptor antagonists, such as esmolol (administered intravenously), landiolol (administered intravenously), or bisoprolol (administered orally). Cardioselective β-blockers offer cardioprotection, prevent atrial fibrillation, and are the recommended option for people with asthma. For people who cannot take β-blockers due to medical reasons, it is recommended to utilize a calcium channel antagonist (such as verapamil or diltiazem) to regulate the ventricular rate [34].

Levothyroxine treatment is cost-effective and simple to administer, making it the preferred therapy for hypothyroidism. Treatment with levothyroxine enhances left ventricular contractility and function, endothelial integrity, and lipid profile, together with cardiac mitochondrial function, according to recent clinical research [11]. When considering levothyroxine for individuals with grade 2 SCHypo (TSH > 10 mIU/L) in younger patients (65 to 70 years old), it is important to examine the patients’ life expectancy and any coexisting conditions. A “start low, go slow” strategy is recommended for starting therapy. This means starting with a low dosage of LT4 (12.5 to 25 μg/day) and progressively increasing it by 12.5 to 25 μg/day every 4 to 8 weeks [16]. For individuals over the age of 80, it is recommended to start therapy with an initial dosage of 20–25 μg. Individuals with pre-existing cardiovascular disease and hypothyroidism should start therapy with a conservative dose of levothyroxine (12.5 μg orally per day) and progressively increase the dosage after six weeks. The goal is to achieve a euthyroid state while avoiding an increase in peripheral vascular resistance, hence improving myocardial reperfusion [8].

Moreover, the administration of TH replacement treatment facilitated the restoration of the neuroendocrine system, resulting in a significant reduction in catecholamines and aldosterone levels in the circulation. This treatment also enhanced cardiac function, as shown by an increase in the volume of blood ejected by the left ventricle. TH replacement treatment may enhance elevated cholesterol levels, rectify myocardial dysfunction, and provide cardiovascular protection [11]. Levothyroxine treatment proved beneficial for people with SCHypo with TSH levels over 10 mIU/L and positive thyroid antibodies. This intervention decreased the incidence of cardiovascular events and improved the quality of life [8]. The therapy summary for SCHyper and SCHypo, according to latest standards, is shown in Table 3.

**Table 3:** Treatment guidelines for subclinical hyperthyroidism and subclinical hypothyroidism.

**Guidelines**	**Consider to treat**	**Consider to observe**
**Subclinical Hyperthyroidism**		
European Thyroid Association (2015) [42]	• TSH 0.1–0.39 mIU/L, age < 65 years, with symptoms TSH 0.1–0.39 mIU/L, age > 65 years, with cardiovascular risk factors or co-morbidity TSH < 0.1 mIU/L, age < 65 years with or without heart disease or comorbidity TSH < 0.1 mIU/L, age > 65 years	• TSH 0.1–0.39 mIU/L or < 0.1 mIU/L, age < 65 years, without symptoms
American Thyroid Association (2016) [19]	• TSH < 0.1 mIU/L or 0.1–0.4 mIU/L, age > 65 years, with symptoms TSH < 0.1 mIU/L or 0.1–0.4 mIU/L, age < 65 years, with symptoms or comorbidities (heart disease, osteoporosis, menopausal)	TSH 0.1–0.4 mIU/L, age < 65 years, without symptoms

**Subclinical Hypothyroidism**		
American Thyroid Association (2012) [43]	• TSH > 10 mIU/L, age < 70 years TSH 4–10 mIU/L, age < 65 years with symptoms	• TSH < 10 mIU/L, age < 70 years TSH 4–10 mIU/L, age > 65 years
European Thyroid Association (2013) [44]	• TSH > 10 mIU/L, age < 70 years TSH < 10 mIU/L, age < 70 years with symptoms TSH > 10 mIU/L, age > 70 with clear symptoms or high cardiovascular risk	• TSH < 10 mIU/L without symptoms, age < 70 years TSH < 10 mIU/L, age > 70 years
National Institute for Health and Care Excellence (NICE) guidelines (2019) [45]	• TSH > 10 mIU/L, age < 70 years TSH 4–10 mIU/L, age < 65 years with symptoms	• TSH > 10 mIU/L, age > 70 years TSH 4–10 mIU/L, age > 65 years

## 8 Conclusion

Thyroid hormone (TH) is essential for the proper structure and function of various human tissues, including the cardiovascular system. Thyroid dysfunction (hypothyroidism and hyperthyroidism) may induce changes in cardiac and endothelial function, plaque formation, dyslipidemia, and high blood pressure, all of which can lead to an increased risk of developing CVD. The use of amiodarone, an antiarrhythmic drug, requires thyroid function monitoring since it may induce thyroid dysfunctions. Every CVD patient is recommended to be screened and treated for any possible thyroid dysfunction to reduce the patient’s mortality and morbidity.

## CRediT authorship contribution statement

**Nanny Natalia Mulyani Soetedjo:** Writing – review & editing, Writing – original draft, Visualization, Supervision, Conceptualization. **Dessy Agustini:** Writing – review & editing, Writing – original draft, Visualization, Conceptualization. **Hikmat Permana:** Writing – review & editing, Writing – original draft.

## Declaration of competing interest

The authors declare that they have no known competing financial interests or personal relationships that could have appeared to influence the work reported in this paper.
